# circ_0038718 promotes colon cancer cell malignant progression via the miR-195-5p/Axin2 signaling axis and also effect Wnt/β-catenin signal pathway

**DOI:** 10.1186/s12864-021-07880-z

**Published:** 2021-10-27

**Authors:** Haitao Gu, Zhiquan Xu, Jianbo Zhang, Yanbing Wei, Ling Cheng, Jijian Wang

**Affiliations:** 1 Department of Gastrointestinal Surgery, Second Affiliated Hospital of Chongqing Medical University, 288 Tianwen Dadao Road, Nanan District, Chongqing, 401336 China; 2 Shanghai Engineering Research Center of Pharmaceutical Translation, Shanghai, 200231 China

**Keywords:** colon cancer, circ_0038718, Wnt/β-catenin signaling pathway, Proliferation, Migration, Invasion

## Abstract

**Objective:**

Colon cancer (CC) is one of the most common cancers whose progression is regulated by a number of factors, including circular RNAs (circRNAs). Nonetheless, circ_0038718 is a novel circRNA, and its regulatory mechanism in CC remains unclear.

**Methods:**

Real-time quantitative PCR (qRT-PCR) was performed to detect the expression of circ_0038718, miR-195-5p and Axin2. Western blot was conducted to determine the protein expression of Axin2 and the key proteins on Wnt/β-catenin signaling pathway. Oligo (dT) _18_ primers and RNase R were employed to identify the circular features of circ_0038718, and the location of circ_0038718 in cells was detected via nucleocytoplasmic separation. Dual-luciferase reporter assay and RNA binding protein immunoprecipitation experiment were carried out to investigate the molecular mechanism of circ_0038718/miR-195-5p/Axin2. Additionally, MTT assay was conducted to assess cell proliferation; Transwell assay was performed to evaluate cell migration and invasion, respectively. The effect of circ_0038718 on CC tumor growth was tested through tumor formation in nude mice.

**Results:**

circ_0038718 was highly expressed in CC and could sponge miR-195-5p in cytoplasm. Silencing circ_0038718 suppressed the proliferative, migratory and invasive abilities of CC cells, while the promoting effect of high circ_0038718 expression on CC cells was reversed upon miR-195-5p over-expression. Axin2 was a downstream target of miR-195-5p and could regulate the Wnt/β-catenin signaling pathway. Axin2 expression was modulated by circ_0038718/miR-195-5p. Knockdown of Axin2 could also attenuate the promoting effect of high circ_0038718 expression on CC cell malignant progression, thus inhibiting tumor growth.

**Conclusion:**

circ_0038718 is able to facilitate CC cell malignant progression via the miR-195-5p/Axin2 axis, which will provide a new idea for finding a novel targeted treatment of CC.

**Supplementary Information:**

The online version contains supplementary material available at 10.1186/s12864-021-07880-z.

## Introduction

Carcinoma of colon and rectum is one of the most common cancers threatening men and women [1]. The incidence of colon cancer (CC) ranks 3rd concerning digestive tract cancers in China [2]. CC pathogenesis is complicated, and the major therapies for CC currently include surgical resection, chemotherapy, radiotherapy and other comprehensive therapies [3]. Despite the fact that the five-year mortality rate of CC has slightly dropped in recent decades [4], the curative effect of the above therapies on advanced CC sufferers remains unsatisfactory [5]. Additionally, as people’s lifestyle and diet structure change, newly developed CC cases and younger patients are rising year by year [2]. Accordingly, there is an urgent need for identifying novel prognostic factors and potential therapeutic targets for CC.

As non-coding RNAs (ncRNAs), circular RNAs (circRNAs) have a unique covalently closed structure [6]. circRNAs play multiple significant roles in cellular physiology functioning as miRNA sponges, transcriptional regulators, RBP binding molecules, templates for protein translation, and immune regulators [7]. It’s worth noting that cancer progression has been confirmed to be associated with dysregulation of circRNAs [8–10]. For instance, Li T et al. [11] suggested that circ_0044516 fosters the proliferation and metastasis of prostate cancer cells as an underlying biomarker. Li J et al. [8] reported that circ_0000711 plays an important role in colorectal cancer (CRC) carcinogenesis and is likely to be a potential and effective biomarker for the diagnosis and prognosis of CRC. In the present study, we discovered that circ_0038718 was markedly differentially expressed in CC through analyzing CC data from the GEO database, but the functional mechanism of circ_0038718 in CC needs further exploration.

MicroRNAs (miRNAs) are small and non-coding RNA molecules whose expression is closely related to cancer progression [12, 13]. miRNAs are involved in the post-transcriptional regulation of gene expression by either inducing mRNA degradation or inhibiting translation by binding to the 3′-untranslational region (3′-UTR) or 5′-UTR of target mRNAs [14]. For example, Jin W et al. [15] demonstrated that miR-671-5p acts as an oncogene of CC and fosters CC cell proliferation, migration and invasion by targeting TRIM67. Cheng B et al. [16] revealed that LINC00662 over-expression can competitively bind to miR-340-5p, making miR-340-5p lowly expressed in CC, thus modulating CLDN8/IL22 co-expression and facilitating the occurrence and development of CC. Research uncovered that circRNAs directly regulate the expression of other RNAs (e.g. miRNAs) via complementary base pairing and can participate in competing endogenous RNA (ceRNA) regulatory networks as a competitive inhibitor of miRNA [17]. miR-195-5p is poorly expressed in various cancers and has been proven to be involved in the regulation of multiple cancers, such as breast cancer, CRC, non-small cell lung cancer (NSCLC) [18–20]. In this study, we found that circ_0038718 could competitively bind to miR-195-5p, but the specific mechanism required further investigation.

The present research aimed to explore the functions of circ_0038718 in CC and the novel mechanism by which circ_0038718 modulates the malignant progression of CC, so as to provide a reference for circ_0038718 to become a diagnostic biomarker of CC. Overall workflow of this study was presented as supplement data (Fig. S1).

## Materials and methods

### Bioinformatics analysis

Bioinformatics studies on the relationship between circRNA, miRNA and mRNA were mainly conducted on the basis of the previous study [21]. The chip sequencing datasets of circRNA, miRNA and mRNA GSE126092, GSE126093 and GSE126094 were downloaded from GEO database. GSE126092 (mRNA) (normal: *n* = 10, tumor: n = 10) /GSE126093 (miRNA) (normal: n = 10, tumor: n = 10) /GSE126094 (circRNA) (normal: n = 10, tumor: n = 10) Series Matrix File (s) were accessed from the Gene Expression Omnibus (GEO) database. Differential analysis was conducted to screen the differentially expressed circRNAs/miRNAs/mRNAs (DE_circRNAs/DE_miRNAs/ DE_mRNAs) using the “limma” package (|logFC| > 2, *p*adj < 0.05). The target circRNA was found to exist in IL4R gene via the circBase database (http://www.circbase.org/cgi-bin/getseq.cgi) and the target miRNAs of circ_0038718 were obtained through the starBase database (http://starbase.sysu.edu.cn/), which were then intersected with the down-regulated DE_miRNAs in the GEO dataset to obtain the target miRNA. Thereafter, miRDB (http://mirdb.org/), TargetScan (http://www.targetscan.org/vert_71/) and miRTarBase (http://mirtarbase.mbc.nctu.edu.tw/php/index.php) were employed to obtain all the target genes of miR-195-5p, which were then intersected with the up-regulated DE_mRNAs to obtain the potential target gene.

### Clinical sample collection

Between January 2019 and June 2020, tumor tissue and matched adjacent tissue from 20 patients suffering from colorectal cancer in Second Affiliated Hospital of Chongqing Medical University. The sampling procedure was carried out during operation, and the adjacent tissue was sampled 2 cm away from the edge of the cancerous tissue. All related patients admitted to receiving no radiotherapy or chemotherapy before the operation, and all samples were with a confirmed diagnosis from pathologists of rich experience. After sampling, tissue samples were transferred into RNA preservation solution at once. The sampling had obtained the agreement and the authorization of the Ethics Committee of Second Affiliated Hospital of Chongqing Medical University, and all participants signed the Informed Consent. Clinical characteristics were shown in Table 1.

**Table 1:** Characteristic of patients

Characteristics	No. of case (%)
Age (Years)	
≥60	8 (40%)
< 60	12 (60%)
Gender	
Male	9 (45%)
Female	11 (55%)
Size (cm)	
< 5	11 (55%)
≥5	9 (45%)
AJCC stage	
I/II	12 (60%)
III/IV	8 (40%)

### Cell culture

Normal human colon cell line CCD-18Co (BNCC337724) and CC cell lines LoVo (BNCC338601), HT-29 (BNCC100164), HCT116 (BNCC337692) and SW480 (BNCC100604) were purchased from BeNa Culture Collection (BNCC; Beijing, China) and cultured in Dulbecco’s Modified Eagle’s Medium (DMEM; Gibco, Thermo Fisher Scientific, Inc., Waltham, MA, USA) supplemented with 10% fetal bovine serum (FBS; Hyclone; GE Healthcare Life Sciences, Logan, UT, USA), and 100 U/mL penicillin/ streptomycin (Gibco; Thermo Fisher Scientific, Inc.). All the cells were incubated in a constant-temperature incubator of 5% CO_2_ at 37 °C. The mediums were replaced every 2 or 3 d. Mutations of the two cell lines HT29 and SW480 mainly used in this paper are shown in Tables S1-S2.

### Cell transfection

miR-195-5p mimic and mimic NC were obtained from GenePharma (Shanghai, China). Lentivirus packaging Vectors pcDNA3-circ_0038718 were used to overexpress circ_0038718, and si-circ_0038718 was used to knock down circ_0038718. Plasmids oe-Axin2 were used to overexpress Axin2, and the plasmids sh-Axin2 carrying short hair clip RNA was used to knock down the expression of Axin2. Their corresponding negative controls were obtained from Invitrogen (Carlsbad, CA, USA). When HT-29 and SW480 cell lines reached the logarithmic phase, cell suspension (5 × 10^4^ cells/well) was inoculated into 6-well plates and maintained in fresh medium. Subsequently, miR-195-5p mimic/mimic NC and lentivirus vectors were transfected into HT-29 and SW480 cells using Lipofectamine 2000 (Thermo Fisher Scientific, Inc.) according to the manufacturer’s instructions and then the cells were incubated in corresponding medium of 5% CO_2_ at 37 °C. All cells were cultured in complete medium for at least 24 h before transfection and rinsed with phosphate-buffered saline (PBS, pH 7.4) before transient transfection.

### Real-time quantitative PCR (qRT-PCR)

Total RNA was extracted from cells using TRIzol reagent (Invitrogen) following the manufacturer’s protocol and RNA concentration was determined by NanoDrop 2000 (Thermo Fisher Scientific, Inc.). Then, RNA was transcribed into complementary DNA (cDNA) by using the Transcriptor Universal cDNA Master (Roche, Basel, Switzerland). qRT-PCR was performed on ABI 7500 Real-Time PCR system (Applied Biosystems; Thermo Fisher Scientific, Inc.) using SYBR Green Kit (Takara, Dalian, China). GAPDH and U6 were used as internal references. The quantitative value was expressed using the 2^-ΔΔCt^ method. The primers were listed in Table 2.

**Table 2:** Primer sequences

Genes	Sequence
circ_0038718	F: 5′-GAACACAGAACAGCATAAGAG-3′
	R: 5′-CATTCCCTCCTGTGCTCAAC-3′
IL4R	F: 5′-CGTGGTCAGTGCGGATAACTA-3′
	R: 5′-TGGTGTGAACTGTCAGGTTTC-3′
Axin2	F: 5′-AGCCAAAGCGATCTACAAAAGG-3′
	R: 5′-AAGTCAAAAACATCTGGTAGGCA-3′
GAPDH	F: 5′-TGCACCACCAACTGCTTAGC-3′
	R: 5′-TGCACCACCAACTGCTTAGC-3′
miR-195-5p	F: 5′-ACACTCCAGCTGGGTAGCAGCACAGAAAT-3′
	R: 5′-TGGTGTCGTGGAGTCG-3′
U6	F: 5′-CTCGCTTCGGCAGCACA-3′
	R: 5′-AACGCTTCACGAATTTGCGT-3′

### circRNA identification

We conducted 2 experiments to identify the circular feature of circ_0038718. Oligo (dT) _18_ primers can only amplify RNAs containing poly-A tails, while random primers can amplify all RNAs. Oligo (dT) _18_ and random primers were employed to identify whether circ_0038718 contains poly-A tails by qRT-PCR. To put it simply, Oligo (dT) 18 and random primers were respectively used to reverse transcribe total RNA, and the total cDNA was then detected by qRT-PCR. If the expression of target gene can be detected in both, it means that it is linear RNA. If the expression of target gene cannot be detected in the reverse transcribed cDNA of Oligo (dT) 18, it means that the RNA has no polydT tail and is a circular RNA. Ribonuclease R (RNase R; Duma, Shanghai, China) is only able to degrade linear RNA but doesn’t affect circRNA. In the experiment, RNase R was first treated at 37 °C for 10 min, and then inactivated in water bath at 70 °C for 10 min. The treated RNA was obtained for reverse transcription, and the expression of target RNA was detected by qPCR. After the extracted RNA was processed by RNase R, qRT-PCR was performed to identify the circular feature of circ_0038718.

### MTT assay

HT-29 and SW480 cell suspension (5 × 10^4^ cells/well) was inoculated into 96-well plates. At 0 h, 24 h, 48 h and 72 h, 10 μL of MTT solution (5 mg/mL) was added into each well, and then the cells were incubated at 37 °C for 4 h. Thereafter, the supernatant was removed and 200 μL dimethyl sulfoxide (DMSO) was added to dissolve the formed formazan crystals. At last, the absorbance of each well at 490 nm was measured by using the Model 680 Microplate Reader (Bio-Rad Laboratories, Inc., Hercules, CA, USA).

### Transwell assay

Cell migration and invasion were evaluated via Transwell assay. Approximately 5 × 10^4^ HT-29 and SW480 cells were seeded into the upper chamber of a 24-well plate inserted with transwell inserts coated with (invasion assay) or without (migration assay) Matrigel (BD Biosciences, San Jose, CA, USA), while the lower chamber was filled with DMEM supplemented with 10% FBS to stimulate cell migration and invasion. After cells were cultured in an incubator of 5% CO_2_ at 37 °C for 36 h, a cotton swab was used to remove non-invasive and non-migratory cells in the upper chamber, after which cells in the lower chamber were fixed in 4% paraformaldehyde for 30 min and stained with crystal violet. After the chambers were dried, 4 fields were randomly chosen and cells were counted under a microscope.

### circRNA localization

For localization of circRNA, the cytoplasmic and nuclear RNA of HT-29 and SW480 cell lines were separated and extracted using the Cytoplasmic & Nuclear RNA Purification Kit (Amyjet). qRT-PCR was performed to determine circ_0038718 expression in the cytoplasm and nucleus. GAPDH and U6 were used as controls of the cytoplasmic and nuclear RNA, respectively. According to the expression ratio of the target gene in the nucleus and cytoplasm, whether it is an intracytoplasmic/nuclear expressed gene was determined.

### Dual-luciferase reporter assay

Dual-luciferase reporter assay was carried out to verify the targeting relationship between miR-195-5p and circ_0038718 or Axin2. Wild type (WT) and mutant (MUT) fragments of Axin2 3′-UTR and circ_0038718 which harbor the binding sites of miR-195-5p were synthesized by GenePharma and sequentially ligated to luciferase reporter vectors pGL3 (Promega, Madison, Wisconsin, USA). The constructed plasmids and miR-195-5p mimic/mimic NC were co-transfected into HEK-293 T cells (ATCC CRL-1573). Cell lysates were collected after 48 h of transfection. The Firefly and Renilla luciferase activities were assessed using the Dual-Luciferase Reporter Assay kit (Promega Corporation).

### RNA binding protein immunoprecipitation (RIP)

RNA binding protein immunoprecipitation (RIP) experiment was carried out using Magna RIP Assay Kit (Millipore, Billerica, MA, USA) according to the manufacturer’s instructions. After being transfected with miR-195-5p mimic/mimic NC for 48 h, HT-29 and SW480 cells were lysed using RIP lysis buffer for 30 min. Thereafter, cell extracts were cultured in RIP buffer containing magnetic beads binding with Ago2 antibody at 4 °C for 4 h, with normal rabbit IgG as negative control. Immunoprecipitated RNA was purified and analyzed via qRT-PCR.

### Western blot

Total proteins were extracted from cells using RIPA lysis buffer (Invitrogen; Thermo Fisher Scientific, Inc.) and the concentration of proteins was measured by bicinchoninic acid (BCA) protein assay kit (Beyotime Institute of Biotechnology, Haimen, China). Protein samples were separated on sodium dodecyl sulphate-polyacrylamide gel electrophoresis (SDS-PAGE) and transferred onto polyvinylidene fluoride (PVDF; Amersham, USA) membranes. After being blocked with 5% non-fat milk at room temperature for 1 h, the membranes were incubated with primary rabbit polyclonal antibodies Axin2 (ab32197, 1:1000, abcam, UK), β-catenin (ab32572, 1:5000, abcam, UK), c-Myc (ab39688, 1:1000, abcam, UK), cyclin D1 (ab16663, 1:200, abcam, UK) and GAPDH (ab9485, 1:2500, abcam, UK) overnight at 4 °C, followed by hybridization with horseradish peroxidase (HRP) -conjugated secondary antibody goat anti-rabbit IgG H&L (ab6721, abcam, UK) at room temperature for 1 h. The membranes were washed with PBST (PBS buffer containing 0.1% Tween-20) in triplicate with 10 min for each time before and after hybridization. Images of the protein bands were observed and captured under an optical luminometer (GE, USA).

### Tumor formation in nude mice

Eighteen female BALB/C nude mice (4–5 weeks old) were derived from National 0Laboratory Animal Center (Beijing, China). The mice were randomly divided into 3 groups with 6 mice in each group, each group were later injected with cancer cells pretreated with si-NC + oe-NC, si-NC + oe-Axin2 and si-circ0038718 + oe-Axin2 respectively. Lentivirus packaging vectors transfected CC cell line SW480 (1 × 10^6^) which described previously was subcutaneously injected into the right lower extremity of nude mice in the 3 groups and tumor growth was monitored and pictures were photographed. Tumor volume was monitored once a week and tumor growth curve was drawn. At the end of the 5th week, mice were given a euthanasia with tumors being isolated. Tumor weight and size were measured and tumor tissue was stored at − 80 °C for further study.

### Immunohistochemistry

Samples were fixed, paraffin embedded and sectioned into 5-μM slices. Subsequently, samples were incubated with primary antibodies Axin2 (ab32197, 1:300, abcam, UK), β-catenin (ab32572, 1:500, abcam, UK), c-Myc (ab39688, 1:100, abcam, UK) and cyclin D1 (ab16663, 1:100, abcam, UK) overnight at 4 °C, followed by hybridization with HRP-conjugated secondary antibody normal rabbit IgG (ab6721, abcam, UK) at 37 °C for 60 min. Finally, the slices were stained with 3,3′-diaminobenzidine (DAB), and the nuclei were counterstained with hematoxylin. Images were captured with an Olympus optical microscope.

### Statistical analysis

All statistical analyses were performed using Graphpad Prism 6.0 (Graphpad Prism 6.0, San Diego, CA, USA) software. All experiments were performed at least in triplicate in order to obtain the mean values. All the measurement data were expressed as mean ± standard deviation (SD), and differences between two groups were analyzed by Student’s *t* test. Pearson correlation analysis was performed to calculate correlations between gene expression levels. *p* < 0.05 was considered statistically significant.

## Results

### circ_0038718 is up-regulated in CC

**Fig. 1:**
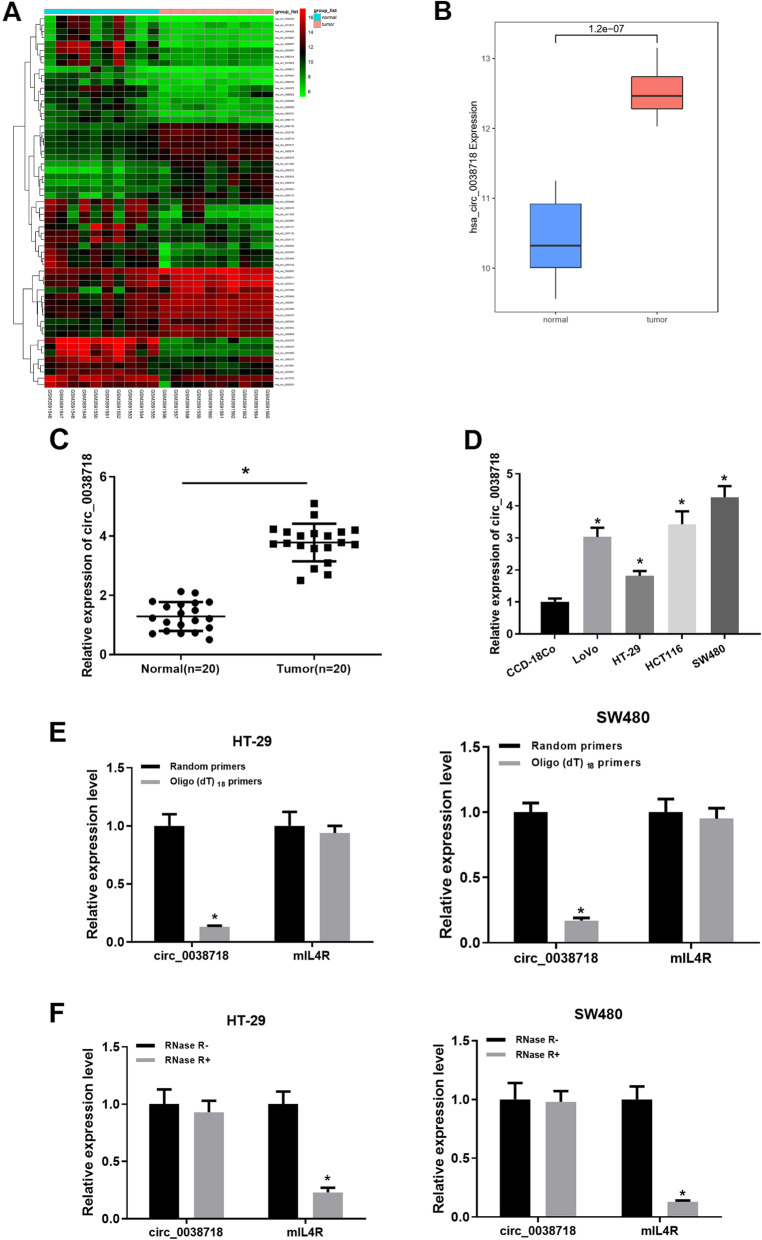
circ_0038718 is up-regulated in CC tissue and cells. (**A**) 59 DE_circRNAs obtained from GSE126094 (circRNA) Series Matrix File; (**B**) Relative expression of circ_0038718 in the GEO database (Blue and red boxes represent normal and tumor group, respectively); (**C**) Expression of circ_0038718 in adjacent tissue and colorectal cancer tissue, respectively; (**D**) qRT-PCR was performed to detect circ_0038718 expression in normal human colon cell line CCD-18Co and CC cell lines LoVo, HT-29, HCT116 and SW480; (**E**) Relative expression of circ_0038718 and mIL4R in HT-29 and SW480 cells were analyzed by qRT-PCR after normalized with random primers and oligo (dT) _18_ primers; (**F**) Relative expression of circ_0038718 and mIL4R in HT-29 and SW480 cells were assessed via qRT-PCR after treatment with RNase R. * *p* < 0.05

GSE126094 (circRNA) Series Matrix File (normal: *n* = 10, tumor: n = 10) was downloaded from the GEO database. Differential analysis was conducted to screen the DE_circRNAs using the “limma” package (|logFC| > 2, *p*adj < 0.05). 59 DE_circRNAs were obtained (Fig. 1A, Table S3), among which circ_0038718 was stably expressed in both normal samples and tumor ones and was most markedly differentially expressed (Fig. 1B). By querying human genome data (GRCh37/hg19), it was found that circ_0038718 is a lncRNA located on chromosome 16, which is mainly composed of exon 3 and exon 4 of IL4R gene (Fig. S2). Furthermore, we detected the expression of circ_0038718 in both adjacent tissue and colorectal cancer tissue, respectively. The result was consistent with the bioinformatics analysis (Fig. 1C).

In order to clarify whether circ_0038718 plays an important role in regulating the occurrence and development of CC cells, we chose normal human colon cell line CCD-18Co and CC cell lines LoVo, HT-29, HCT116 and SW480 for experiments, discovering that circ_0038718 expression in CC cell lines was significantly higher than that in normal human colon cell line (Fig. 1D). Subsequently, we chose CC cell lines HT-29 and SW480 respectively with the lowest expression and the highest expression of circ_0038718 for the following cell experiments. First, we verified the circular feature of circ_0038718 and conducted qRT-PCR using oligo (dT) _18_ and random primers, the result of which suggested that compared with random primers, circ_0038718 expression in HT-29 and SW480 cells was noticeably reduced while mIL4R expression didn’t change when using oligo (dT) _18_ primers, revealing that circ_0038718 contained no poly-A tail (Fig. 1E). Meanwhile, circ_0038718 was resistant to RNase R, but linear RNA mIL4R could be digested by RNase R, which demonstrated that circ_0038718 was circular (Fig. 1F). Collectively, the above results unveiled that circ_0038718 was highly expressed in CC and had a circular structure.

### circ_0038718 can regulate CC cell growth

**Fig. 2:**
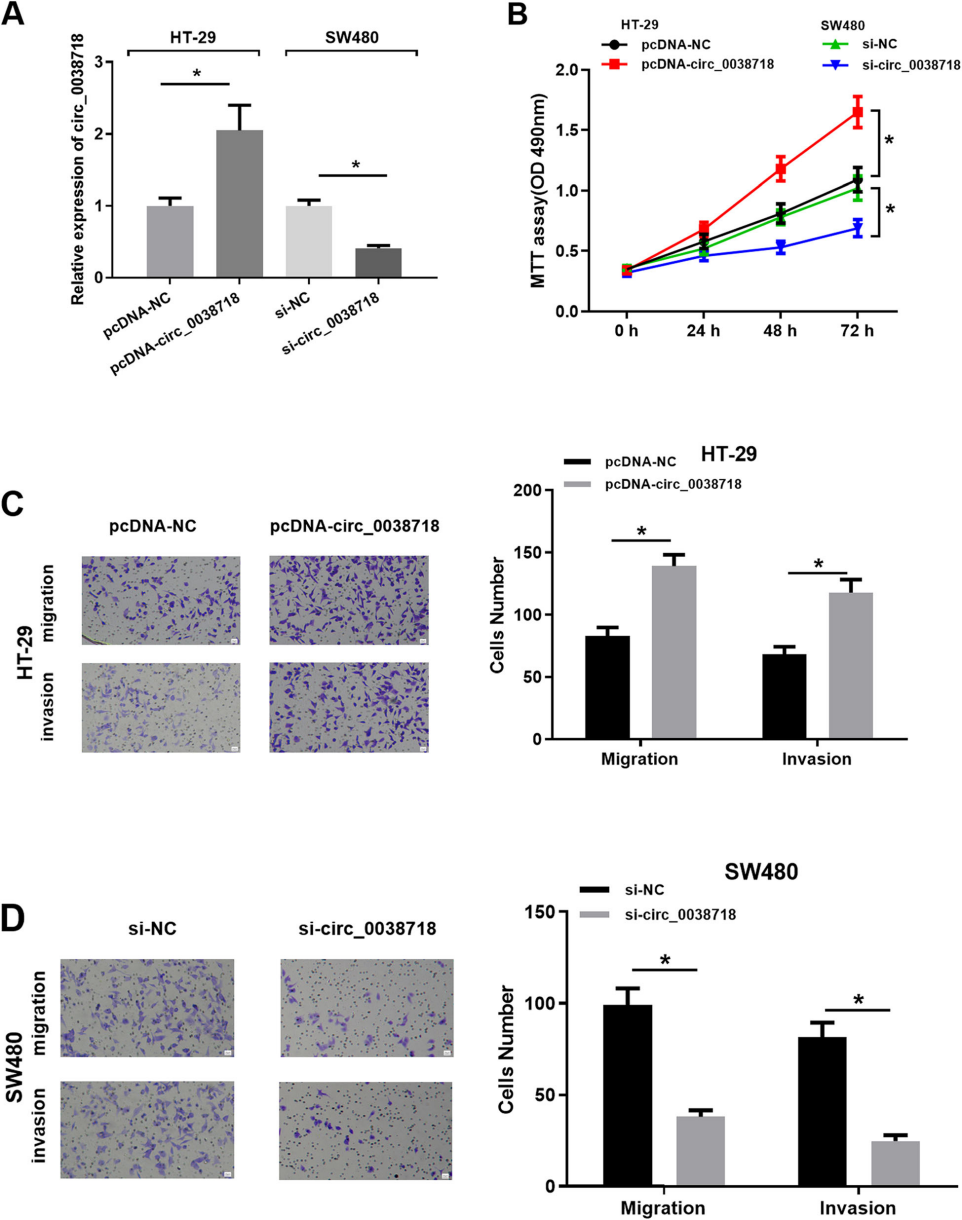
circ_0038718 promotes CC cell proliferation, migration and invasion. (**A**) qRT-PCR was performed to detect circ_0038718 expression in all groups; (**B**) MTT assay was conducted to assess the viability and proliferative ability of CC cells in all groups; (**C**-**D**) Transwell assay was carried out to evaluate CC cell migratory and invasive capacities in all groups (100×). * *p* < 0.05

circ_0038718 was respectively over-expressed and silenced in HT-29 and SW480 cell lines, and qRT-PCR was carried out to test the expression efficiency of circ_0038718 (Fig. 2A), suggesting that the expression efficiency of circ_0038718 was high in the two cell lines, which could be used for follow-up experiments. MTT assay implicated that the proliferative ability of CC cells was promoted upon circ_0038718 over-expression but inhibited upon circ_0038718 silencing (Fig. 2B). Transwell assay showed that CC cell migratory and invasive capacities were fostered by over-expressing circ_0038718 but suppressed by knocking down circ_0038718 (Fig. 2C-D). As a result, the above experiments suggested that high circ_0038718 expression facilitated CC cell proliferation, migration and invasion, where low circ_0038718 expression repressed the malignant progression of CC cells.

### miR-195-5p is a direct target of and negatively regulated by circ_0038718

**Fig. 3:**
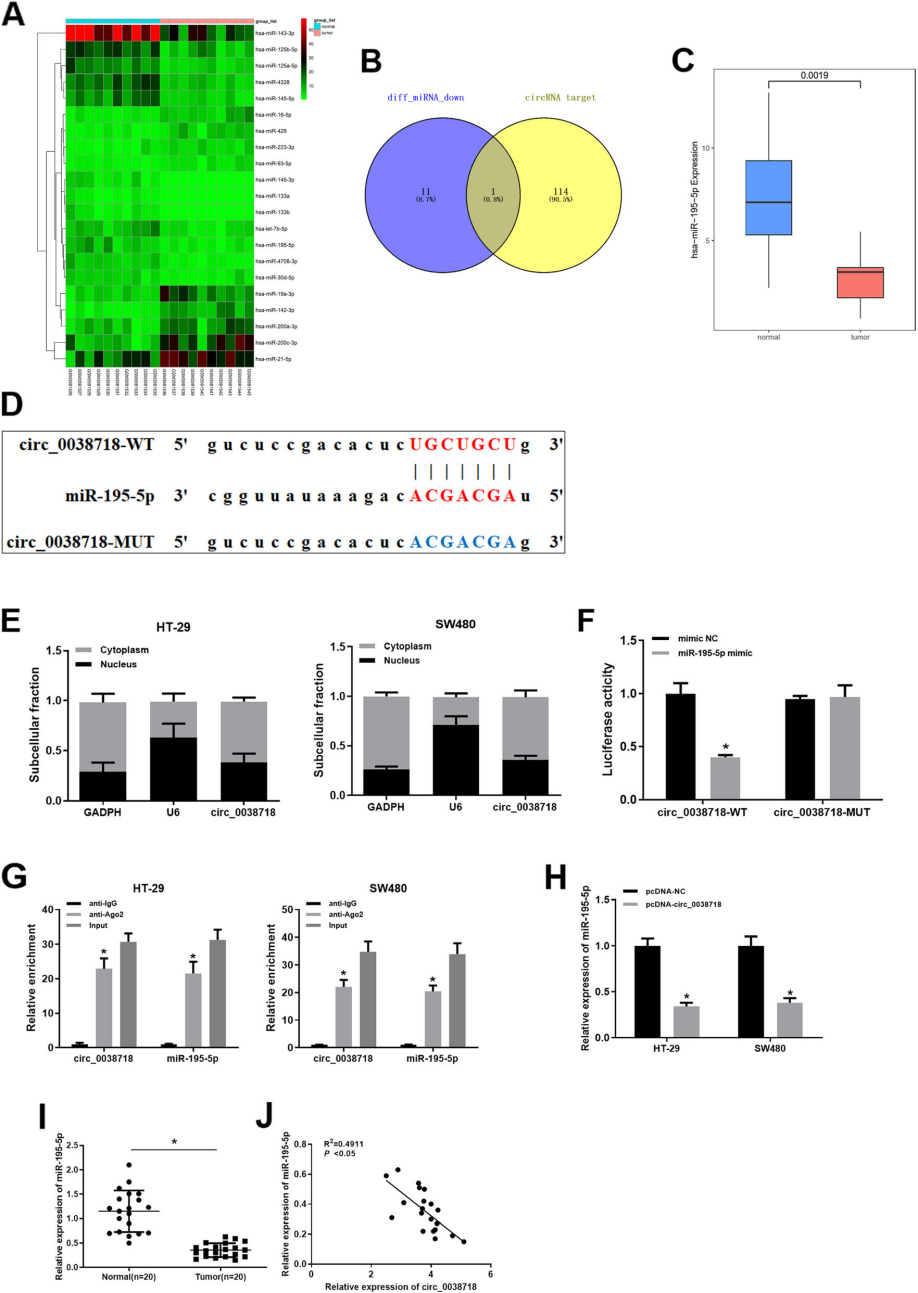
miR-195-5p is a direct target of and negatively regulated by circ_0038718. (**A**) 21 DE_miRNAs obtained from the GSE126093 Series Matrix File; (**B**) 12 down-regulated DE_miRNAs were intersected with 115 predicted target genes of circ_0038718; (C) Relative expression of miR-195-5p in GSE126093 Series Matrix File. Blue and red boxes stand for the normal and tumor groups, respectively; (**D**) Predicted binding site between miR-195-5p and circ_0038718; (**E**) qRT-PCR was conducted to test the expression of circ_0038718, U6 and GAPDH in cytoplasm and nucleus after nucleocytoplasmic separation of HT-29 and SW480 cells; (**F**-**G**) Dual-luciferase reporter assay and RIP experiment were performed to verify the targeting relationship between miR-195-5p and circ_0038718; (**H**) qRT-PCR was used to evaluate the effect of circ_0038718 over-expression on miR-195-5p; (**I**) The expression of miR-195-5p in colorectal cancer tissue and adjacent tissue, respectively; (**J**) The correlation between circ_0038718 and miR-195-5p expression level. * *p* < 0.05

In order to explore the underlying mechanism by which circ_0038718 modulates CC cell malignant progression, we obtained the target genes of circ_0038718 via the starBase database and 21 DE_miRNAs from the GSE126093 Series Matrix File (Fig. 3A, Table S4). After 12 down-regulated DE_miRNAs were intersected with the predicted target genes of circ_0038718, we found that only miR-195-5p might be the potential target of circ_0038718, and miR-195-5p was remarkably down-regulated in CC (Fig. 3B-C). Bioinformatics analysis indicated that there was a binding site between miR-195-5p and circ_0038718 (Fig. 3D). qRT-PCR was employed to determine the subcellular location of circ_0038718, the result of which suggested that circ_0038718 mainly resided in cytoplasm, demonstrating that circ_0038718 was able to interact with miR-195-5p in cytoplasm (Fig. 3E). Dual-luciferase reporter assay and RIP experiment were performed to verify the targeting relationship between miR-195-5p and circ_0038718. In the dual-luciferase reporter assay, over-expressing miR-195-5p reduced the luciferase activity of circ_0038718-WT but showed no effect on that of circ_0038718-MUT (Fig. 3F). The RIP experiment implicated that compared with IgG antibody (control), circ_0038718 and miR-195-5p were highly enriched in the precipitated RNA-Ago2 antibody complex (Fig. 3G). qRT-PCR result uncovered that miR-195-5p was prominently down-regulated in CC cells upon circ_0038718 over-expression (Fig. 3H). Whereafter, we confirmed that miR-195-5p were markedly lower expressed in colorectal cancer tissue than in adjacent tissue (Fig. 3I). Pearson correlation analysis showed a negative correlation between circ_0038718 and miR-195-5p expression level (Fig. 3J). Taken together, the above results unveiled that circ_0038718 could directly bind with miR-195-5p and regulate its expression.

### Up-regulation of miR-195-5p can reduce the promoting effect of circ_0038718 on CC cell proliferation, migration and invasion

**Fig. 4:**
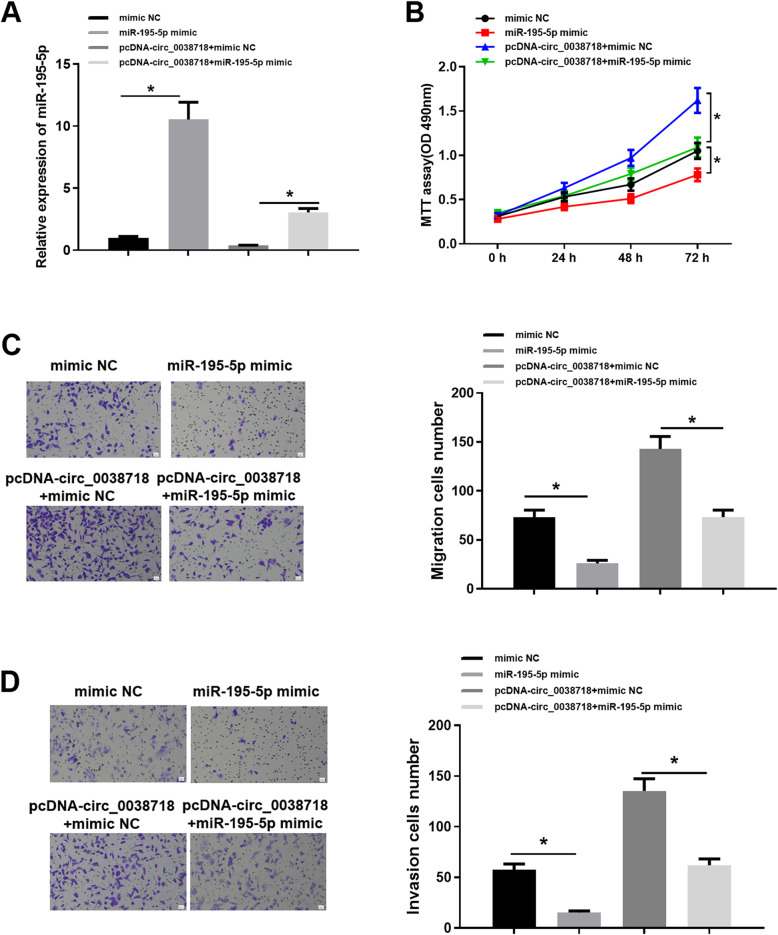
Up-regulation of miR-195-5p can reduce the promoting effect of circ_0038718 on CC cell proliferation, migration and invasion. (**A**) qRT-PCR was performed to detect miR-195-5p expression in all groups; CC cell viability and proliferative ability were assessed via (**B**) MTT assay; (**C**-**D**) Transwell assay was conducted to evaluate CC cell migratory and invasive capacities (100×). * *p* < 0.05

In order to further investigate the effect of circ_0038718 modulating miR-195-5p on CC cells, we carried out a series of in vitro experiments for verification. Firstly, miR-195-5p expression in HT-29 cell line in the mimic NC, miR-195-5p mimic, pcDNA-circ_0038718 + miR-195-5p mimic and pcDNA-circ_0038718 + mimic NC groups was determined, implicating that miR-195-5p was markedly up-regulated after cells were transfected with miR-195-5p mimic (Fig. 4A). Next, MTT assay uncovered that over-expressing miR-195-5p significantly repressed CC cell proliferation and attenuated the promoting effect of circ_0038718 on CC cell proliferative ability (Fig. 4B). Additionally, Transwell assay indicated that the migratory and invasive capacities of CC cells were effectively suppressed after transfection with miR-195-5p mimic, and miR-195-5p over-expression remarkably counteracted the promoting effect of high circ_0038718 expression on CC cell migration and invasion (Fig. 4C-D). Consequently, it could be concluded from the above results that up-regulation of miR-195-5p could reverse the promoting effect of circ_0038718 on CC cell proliferation, migration and invasion.

### miR-195-5p regulates Axin2 expression and could affect Wnt/β-catenin signaling pathway

**Fig. 5:**
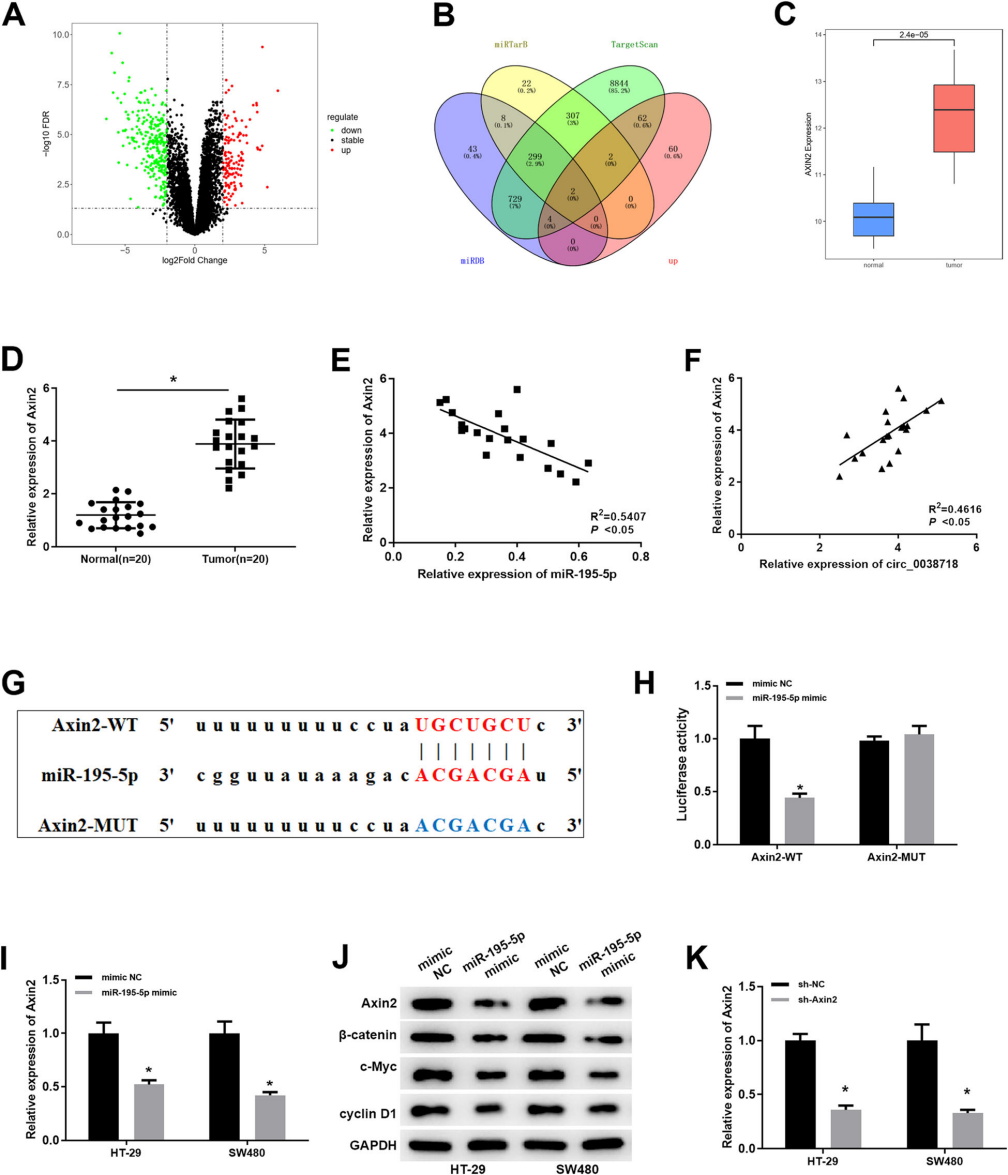
miR-195-5p regulates Wnt/β-catenin signaling pathway by targeting Axin2. (**A**) Volcano plot of 415 DE_mRNAs in the GEO database (Red and green dots represent up-regulated and down-regulated genes, respectively); (**B**) Venn diagram of up-regulated DE_mRNAs in the GEO database and the predicted target mRNAs of miR-195-5p; (**C**) Relative expression of Axin2 in the GEO database (Blue and red boxes represent normal and tumor group, respectively); (**D**) The expression level of Axin2 in colorectal cancer tissue and adjaceent tissue; (**E**) The correlation between Axin2 and miR-195-5p expression level; (**F**) The correlation between Axin2 and circ_0038718 expression level; (**G**) Putative binding sites between miR-195-5p and Axin2; (**H**) Dual-luciferase reporter assay was performed to detect the targeting relationship between miR-195-5p and Axin2; (**I**) qRT-PCR was performed to assess the effect of over-expressing miR-195-5p on Axin2 mRNA; (**J**) Western blot was conducted to evaluate the effect of miR-195-5p over-expression on the protein expression of Axin, β-catenin, c-Myc and cyclin D1; (**K**) qRT-PCR was performed to assess the effect of sh-Axin2 knock down. * *p* < 0.05

In order to further clarify the molecular mechanism of circ_0038718/miR-195-5p, GSE126092 (mRNA) Series Matrix File (normal: *n* = 10, tumor: n = 10) was accessed from the GEO database. Differential analysis was conducted to screen the DE_ mRNAs using the “limma” package (|logFC| > 2, *p*adj < 0.05) and 415 DE_ mRNAs were obtained (Fig. 5A, C, Table S5). Then, miRDB, TargetScan and miRTarBase databases were employed to obtain all the target genes of miR-195-5p, which were then intersected with the up-regulated DE_mRNAs, and 2 potential target genes were obtained: Axin2 and ITGA2 (Fig. 5B). We also verified the expression of Axin2 in colorectal cancer tissue were higher than adjacent tissue (Fig. 5D), and Pearson correlation analysis showed a nagetive correlation between Axin2 and miR-195-5p expression level (Fig. 5E), and a positive correlation between Axin2 and circ_0038718 expression level, respectively (Fig. 5F). Therefore, Axin2 was chosen as the downstream target gene for further study.

According to the predicted binding sites (Fig. 5G), we constructed luciferase reporter vectors Axin2-WT and Axin2-MUT and measured the luciferase activities of different transfection groups, discovering that the luciferase activity of Axin2-WT was noticeably decreased upon miR-195-5p over-expression, whereas that of Axin2-MUT was not markedly affected (Fig. 5H). Meanwhile, Axin2 mRNA and protein expression in HT-29 and SW480 cell lines were significantly reduced, and the expression of β-catenin, c-Myc and cyclin D1 were also down-regulated upon miR-195-5p over-expression (Fig. 5I-J). After the above experiments, to further study the biological function of Axin2 in cells, sh-Axin2 was constructed and its transfection efficiency was verified (Fig. 5K). Collectively, these findings confirmed that miR-195-5p could serve as a negative mediator of the Wnt/β-catenin signaling by targeting to down-regulate Axin2.

### circ_0038718/miR-195-5p/Axin2 axis effect Wnt/β-catenin signaling pathway

**Fig. 6:**
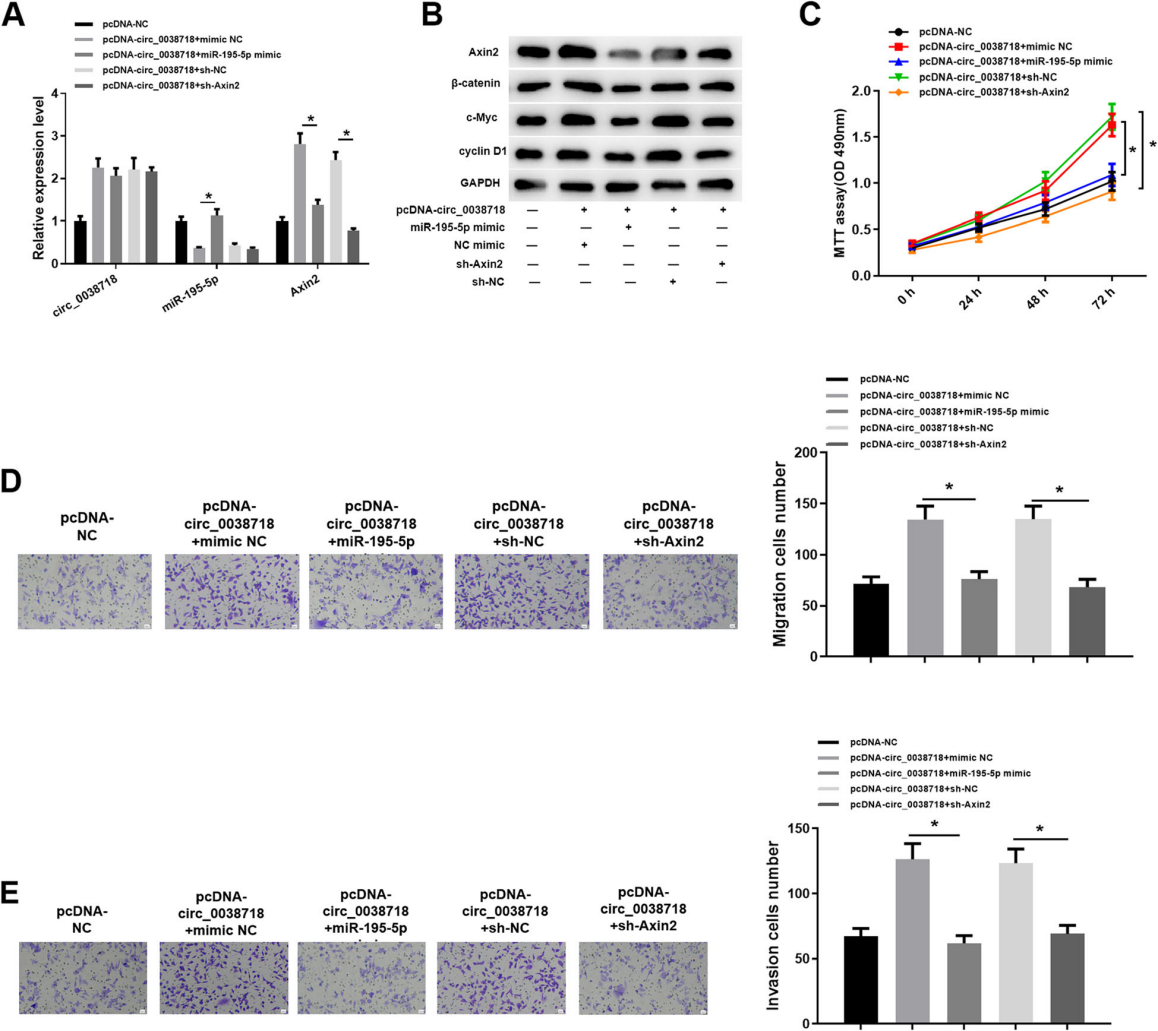
circ_0038718 activates Wnt/β-catenin signaling pathway via the circ_0038718/miR-195-5p/Axin2 axis. (**A**) qRT-PCR was conducted to detect the expression of circ_0038718, miR-195-5p and Axin2 in each group; (**B**) Western blot was performed to determine the protein expression of Axin2 and the key proteins of the Wnt/β-catenin signaling pathway; CC cell viability and proliferative ability were assessed via (**C**) MTT assay; (**D**-**E**) Transwell assay was conducted to evaluate CC cell migratory and invasive capacities (100×). * *p* < 0.05

Since Axin2 is associated with the Wnt/β-catenin signaling pathway, we further explored the possible influence on the Wnt/β-catenin signaling pathway in this study. We set 5 groups: pcDNA-NC, pcDNA-circ_0038718 + mimic NC, pcDNA-circ_0038718 + miR-195-5p mimic, pcDNA-circ_0038718 + sh-NC and pcDNA-circ_0038718 + sh-Axin2. We detected the expression of circ_0038718, miR-195-5p and Axin2 in the 5 groups, finding that Axin2 was up-regulated in the pcDNA-circ_0038718 + mimic NC group but then down-regulated in the pcDNA-circ_0038718 + miR-195-5p mimic group; the promoting effect of circ_0038718 on Axin2 was reversed in the pcDNA-circ_0038718 + sh-Axin2 group (Fig. 6A). Meanwhile, western blot suggested the similar result and the protein expression of β-catenin, c-Myc and cyclin D1 was basically consistent with that of Axin2 (Fig. 6B). Additionally, in vitro experiments demonstrated that in the groups with high Axin2, the proliferative, migratory and invasive abilities of CC cells were relatively stronger, while the cancer-promoting effect of circ_0038718 was reversed in the pcDNA-circ_0038718 + miR-195-5p mimic group and pcDNA-circ_0038718 + sh-Axin2 group (Fig. 6C-E). To sum up, the present study proved that circ_0038718 may affect the Wnt/β-catenin signaling pathway via the circ_0038718/miR-195-5p/Axin2 axis and promote CC cell proliferation, migration and invasion.

### circ_0038718 silencing reduces CC cell tumorigenesis in vivo

**Fig. 7:**
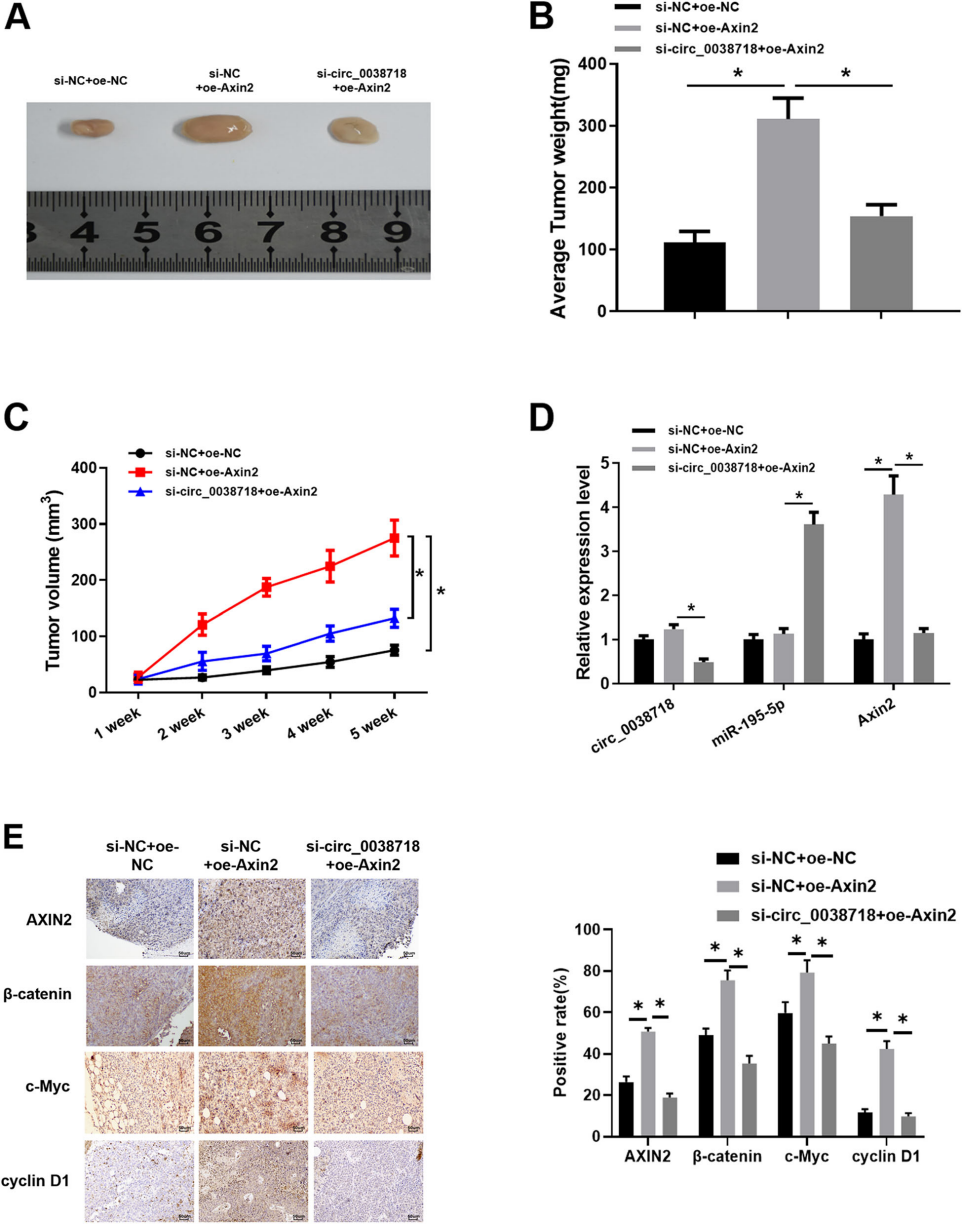
circ_0038718 silencing reduces CC cell tumorigenesis in vivo*.* (**A**) Tumor cell tumorigenesis of nude mice in all groups was identified; (**B**) Average tumor weight of nude mice after 5 weeks; (**C**) Tumor volume of nude mice in all groups for 5 weeks; (**D**) qRT-PCR was performed to detect the expression of circ_0038718, miR-195-5p and Axin2 in tumor tissue of nude mice; (**E**) Immunohistochemistry was conducted to assess the expression of Axin2, β-catenin, c-Myc and cyclin D1 in tumor tissue (400×). * *p* < 0.05

Finally, we carried out tumor formation in nude mice experiment to verify the results of the above experiments. Stably expressed SW480 cell suspension was subcutaneously injected into nude mice, and tumor weight and size were observed. The result unveiled that compared with the control group, tumor volume and weight in the si-NC + oe-Axin2 group were markedly increased, whereas those in the si-circ_0038718 + oe-Axin2 group were significantly decreased (Fig. 7A-C). Next, qRT-PCR was conducted to determine the expression of circ_0038718, miR-195-5p and Axin2 in tumor tissue of nude mice, the result of which uncovered that miR-195-5p was markedly up-regulated while Axin2 was down-regulated upon circ_0038718 silencing (Fig. 7D). Immunohistochemistry also indicated that Axin2 expression was associated with the Wnt/β-catenin signaling pathway. The positive cell rate of β-catenin, c-Myc and cyclin D1 were raised upon Axin2 over-expression, whereas those were significantly reduced after Axin2 was down-regulated caused by circ_0038718 knockdown (Fig. 7E). Taken together, the above results confirmed that Axin2 could activate the Wnt/β-catenin signaling pathway, while circ_0038718 silencing could inhibit the malignant progression of CC cells and the Wnt/β-catenin signaling pathway.

## Conclusion

Currently, the morbidity and mortality of CC sufferers have been on the rise, which has become an important public health issue worldwide [22]. Therefore, clarifying the molecular mechanism affecting CC progression is beneficial to the development of novel clinical therapeutic strategies for CC. Research suggested that cicrRNAs are able to serve as biomarkers of tumors and regulate the progression of multiple cancers [23–25]. Ju HQ et al. [26] uncovered that cicrRNAs can predict post-operative recurrence in stage II/III CC through analyzing 667 cases of R0 resected stage II/III CC. Additionally, Hsiao K.Y. et al. [27] unveiled that CircCCDC66 is highly expressed in polyps and CC and is connected with poor prognosis; high CircCCDC66 expression is capable of fostering CC growth and metastasis. In the present study, we demonstrated that circ_0038718 was up-regulated in CC. Besides, we proved that circ_0038718 was a stable circular transcript, contained no poly-A tail and was resistant to RNase R.

The role of circRNAs in regulating gene expression has attracted increasing attention. For instance, circ_0071589 can act as a molecular sponge for miR-600 to promote EZH2 expression [28]. circ_0136666 facilitates CRC proliferation and invasion via miR-136/SH2B1 axis [29]. circ_0055625 fosters CC cell growth by sponging miR-106b-5p^10^. In this study, we validated that up-regulation of circ_0038718 promoted CC cell proliferation, migration and invasion, while its down-regulation produced opposite result, revealing that circ_0038718 expression plays a crucial role in the normal biological functions of CC cells. As a consequence, circ_0038718 might become a significant target for the diagnosis and treatment of CC.

In order to clarify the molecular mechanism of circ_0038718 as a ceRNA in CC cells, the predicted target genes of circ_0038718 were intersected with the down-regulated DE_miRNAs in the GEO database, showing that miR-195-5p was highly likely to be a potential target of circ_0038718. Luo Q et al. [30] reported that miR-195-5p is noticeably down-regulated in CC cells, and miR-195-5p over-expression could regulate cell growth and cycle through targeting CDK8. Sun M et al. [31] suggested that miR-195-5p is remarkably down-regulated in CC cells and inhibits tumor growth by silencing YAP1. Numerous studies implicated that miR-195-5p suppresses the malignant progression of CC, but there has been still no investigation on whether miR-195-5p could interact with circ_0038718. In our study, we confirmed that circ_0038718 chiefly located in the cytoplasm, which provided theoretical support for that circ_0038718 sponged miR-195-5p in the cytoplasm. Meanwhile, we validated the targeting relationship between circ_0038718 and miR-195-5p via dual-luciferase reporter assay and RIP experiment, proving that circ_0038718 could down-regulate miR-195-5p in CC cells. In addition, MTT and transwell assay also verified that miR-195-5p could repress the promoting effect of circ_0038718 on CC cell proliferation, migration and invasion. To sum up, the present research unveiled that circ_0038718 could affect CC cell proliferation, migration and invasion by down-regulating miR-195-5p.

In view of the fact that miR-195-5p generally modulates cancer cells by targeting its downstream mRNA, miRDB, TargetScan and miRTarBase databases were employed to obtain all the target genes of miR-195-5p, which were then intersected with the up-regulated DE_mRNAs in the GEO database, and ultimately Axin2 was found to be up-regulated in CC. In the meantime, a series of experiments identified the targeting relationship between miR-195-5p and Axin2 and it was proved that miR-195-5p could target to silence Axin2.

Former research indicated that Axin2 is a direct target of the Wnt signaling pathway and restricts the duration and/or intensity of the Wnt signaling via a negative feedback loop [32]. Besides, Yu J et al. [33] revealed that Axin2 is an important regulatory factor of the Wnt/β-catenin signaling pathway, while CDX2 inhibits the proliferation and tumor formation of CC cells by suppressing the Wnt/β-catenin signaling via transactivation of GSK-3β and Axin2 expression. In our study, a series of experiments and bioinformatics background showed that Axin2 was positively correlated with the expression of beta-catenin, which was inconsistent with the traditional research results (Fig. S3). We suspected that there were other genes or pathways in this regulatory relationship that influenced the regulation of beta-catenin by Axin2. In fact, genes such as Axin2 and miR-195-5p are multi-target in colon cancer, and the overexpression/manipulation of them may affect the expression of other genes, thus resulting in the expression of beta-catenin inconsistent with the common regulatory relationship. Actually, many studies found that Axin2 expression is positively correlated with beta-catenin. For example, in the study of Zhang et al. [34], TMED3 not only promotes the expression of Axin2 but also boosts the expression of beta-catenin. Xia et al. [35] also found in their study that the expression of Axin2 is positively correlated with the expression of beta-catenin. Based on the above studies, we uncovered that beta-catenin was positively regulated by Axin2 in colon cancer, but its internal mechanism still needs further study. In vivo and in vitro experiments demonstrated that silencing Axin2 significantly reversed the promoting effect of circ_0038718 over-expression on CC cell malignant progression. These results all proved that the circ_0038718/miR-195-5p/Axin2 axis plays a significant role in regulating CC progression.

As biomarkers of tumors, many circRNAs have been identified as being associated with the occurrence and development of tumors. circ_0038718 is a novel circRNA, the exploration in mechanism of which will provide a theoretical basis for further investigating the molecular mechanism underlying CC progression and carrying out targeted therapy for CC. In the meantime, our studies will provide a valuable reference for studying the functional mechanism of circ_0038718 in other tumors.

## Supplementary Information

**Additional file 1: Fig. S1.** Overall flowchart of study.

**Additional file 2: Fig. S2.** Genetic information of circ_0038718.

**Additional file 3: Fig. S3.** GEPIA database shows expression status of Axin2 and beta-catenin in COAD (* *p* < 0.05).

**Additional file 4: Table S1.** Mutation landscape of colon cancer cell HT29.

**Additional file 5: Table S2.** Mutation landscape of colon cancer cell SW480.

**Additional file 6: Table S3.** Differential expression analyze result of circRNA.

**Additional file 7: Table S4.** Differential expression analyze result of miRNA.

**Additional file 8: Table S5.** Differential expression analyze result of mRNA.

## Data Availability

The data used to support the findings of this study are included in supplementary table-Raw Data. The data and materials in the current study are available from the corresponding author on reasonable request.
