# Supplementary figures and images for: circ_0038718 promotes colon cancer cell malignant progression via the miR-195-5p/Axin2 signaling axis and also effect Wnt/β-catenin signal pathway
Source: BMC Genomics. 2021 Oct 27;22:768. doi: 10.1186/s12864-021-07880-z (PMC8555003; doi:10.1186/s12864-021-07880-z)

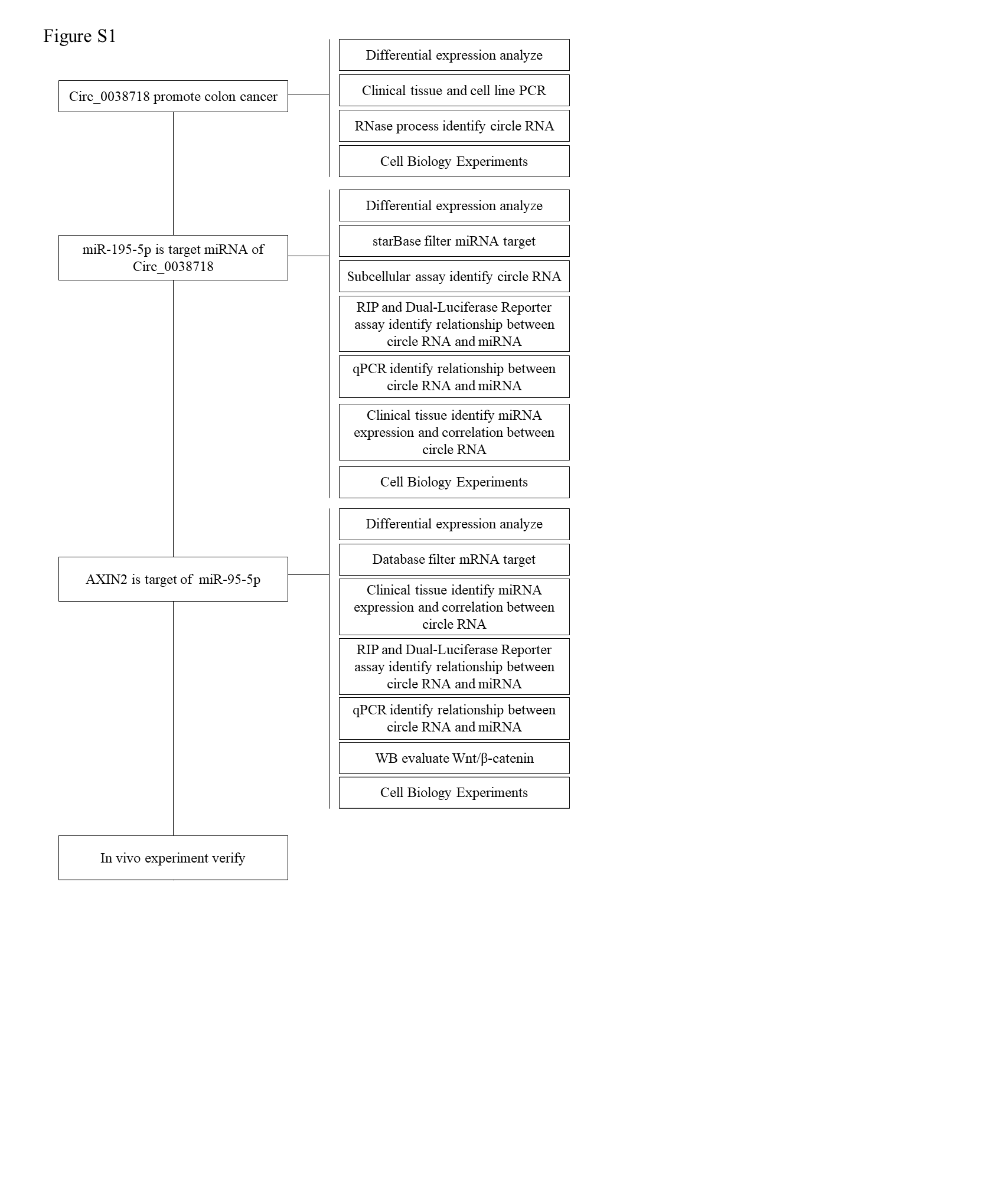

Supplement: Supplementary file 1 — Additional file 1: Fig. S1. Overall flowchart of study. [file 12864_2021_7880_MOESM1_ESM.tif]

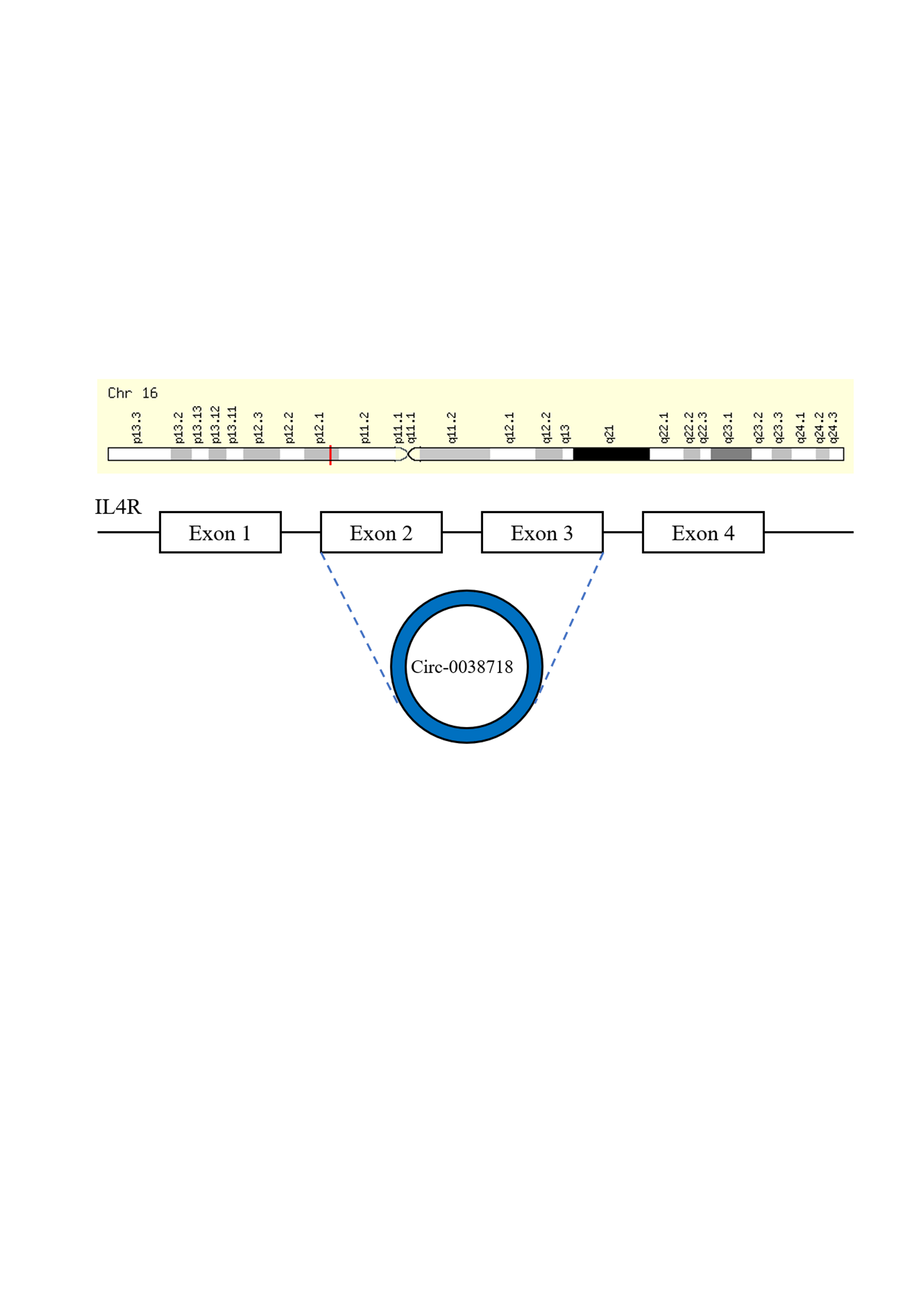

Supplement: Supplementary file 2 — Additional file 2: Fig. S2. Genetic information of circ_0038718. [file 12864_2021_7880_MOESM2_ESM.tif]

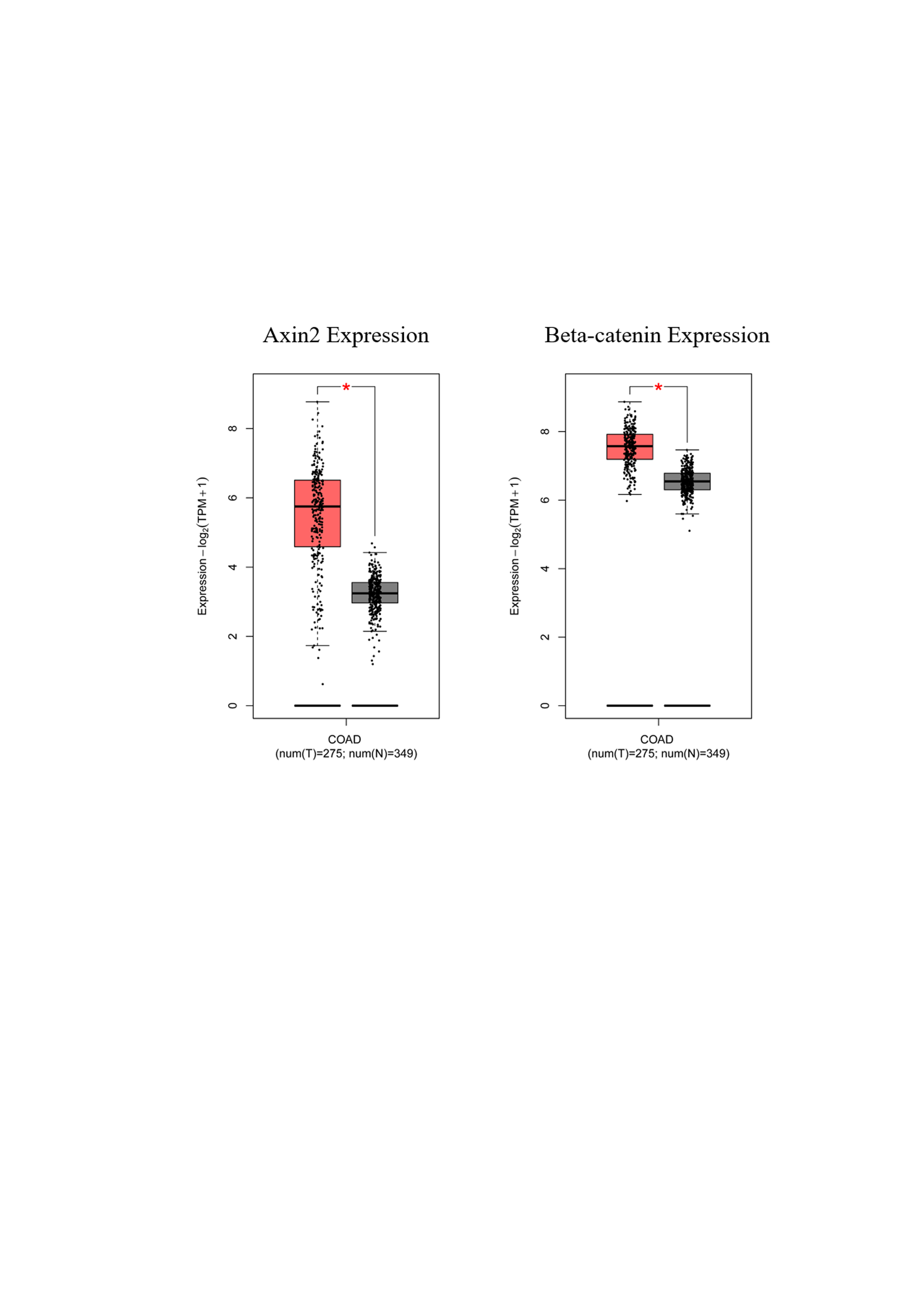

Supplement: Supplementary file 3 — Additional file 3: Fig. S3. GEPIA database shows expression status of Axin2 and beta-catenin in COAD (* p < 0.05). [file 12864_2021_7880_MOESM3_ESM.tif]
